# Shotgun Sequencing Analysis of *Trypanosoma cruzi* I Sylvio X10/1 and Comparison with *T. cruzi* VI CL Brener

**DOI:** 10.1371/journal.pntd.0000984

**Published:** 2011-03-08

**Authors:** Oscar Franzén, Stephen Ochaya, Ellen Sherwood, Michael D. Lewis, Martin S. Llewellyn, Michael A. Miles, Björn Andersson

**Affiliations:** 1 Science for Life Laboratory, Department of Cell and Molecular Biology, Karolinska Institutet, Stockholm, Sweden; 2 Department of Pathogen Molecular Biology, Faculty of Infectious and Tropical Diseases, London School of Hygiene and Tropical Medicine, London, United Kingdom; Lancaster University, United Kingdom

## Abstract

*Trypanosoma cruzi* is the causative agent of Chagas disease, which affects more than 9 million people in Latin America. We have generated a draft genome sequence of the TcI strain Sylvio X10/1 and compared it to the TcVI reference strain CL Brener to identify lineage-specific features. We found virtually no differences in the core gene content of CL Brener and Sylvio X10/1 by presence/absence analysis, but 6 open reading frames from CL Brener were missing in Sylvio X10/1. Several multicopy gene families, including DGF, mucin, MASP and GP63 were found to contain substantially fewer genes in Sylvio X10/1, based on sequence read estimations. 1,861 small insertion-deletion events and 77,349 nucleotide differences, 23% of which were non-synonymous and associated with radical amino acid changes, further distinguish these two genomes. There were 336 genes indicated as under positive selection, 145 unique to *T. cruzi* in comparison to *T. brucei* and *Leishmania*. This study provides a framework for further comparative analyses of two major *T. cruzi* lineages and also highlights the need for sequencing more strains to understand fully the genomic composition of this parasite.

## Author Summary

Chagas disease is a major health problem in Latin America and it is caused by the protozoan parasite *Trypanosoma cruzi*. The genome sequence of the *T. cruzi* strain CL Brener (TcVI) has revealed a genome with large repertoires of genes for surface antigens, among other features. In the present study, we sequenced the genome of a representative member of TcI, the predominant agent of Chagas disease North of the Amazon and performed comparative analyses with CL Brener. Genetic variation between strains can potentially explain differences in disease pathogenesis, host preferences and aid the identification of drug targets. Our analysis showed that the two genomes have very similar sets of genes, but contain large differences in the relative size of several important gene families. Moreover, an abundance of allelic sequence variation was found in a large fraction of genes, and an evolutionary analysis indicated that many genes have evolved at different rates.

## Introduction

The protozoan parasite *Trypanosoma cruzi*, causative agent of Chagas disease, infects 7.7 million people in Latin America and causes 12,500 deaths annually [1]. Transmission of the parasite most commonly occurs if infected faeces of the haematophagous triatomine insect vector makes contact with mucosae or abraded skin. Most morbidity is associated with the chronic stage of the disease, which can take several years to develop. There is no vaccine against *T. cruzi* infections and drug treatment is restricted to a small number of drugs with insufficient efficacy and potentially harmful side effects.

Multiple genotyping strategies support the subdivision of *T. cruzi* into six major phylogenetic groups, recently renamed discrete typing units (DTUs) I-VI by international consensus [2]. DTU distribution can be loosely defined by several parameters including ecology, vector and host preference, geography and disease association [3], although patchy sampling precludes definitive associations. Likewise, an accumulating number of *in vitro* and *in vivo* experiments indicate significant phenotypic variation between *T. cruzi* strains in terms of physiology, biochemistry and infectivity [4], [5], [6], [7], [8], [9], [10], [11], [12], [13]. Again, however, there are few clear-cut correlations between genetic groups and pathogenic potential and the genetic determinants of such differences remain enigmatic. Genome sequencing can provide crucial data to facilitate such research.

TcI is the predominant agent of Chagas disease in the Americas North of the Amazon e.g. [14] [15] [16], although it is by no means uncommon in patients in other regions (e.g. [17]). In contrast, TcII, TcV and TcVI are the predominant causes of Chagas disease in the Southern Cone countries, where megaoesophagus and megacolon are more common [18], [19], [20], [21], [22], [23], [24], [25]. TcI shows spectacular abundance among wild hosts and vectors throughout the endemic range of *T. cruzi*, especially, but not exclusively, in association with *Didelphis* sp. opossums [3], [26]. Whereas the other strains responsible for most human disease, TcII, V and VI, are rarely isolated from natural reservoirs or triatomines. Indeed, minimal diversity across multiple markers in putative TcII/TcIII hybrids TcV and TcVI, and their widespread southerly distribution, are consistent with a recent origin alongside domestic transmission cycles (Lewis et al, submitted). In phylogenetic terms TcI and TcII are most divergent and nucleotides models estimate their MRCA at 3-16 MYA [27]. Concurrent with substantial intraspecific genetic diversity, Chagas disease is characterized by a highly variable clinical presentation [1]. This has long been assumed to be, at least in part, a product of genetic differences between strains of *T. cruzi* [15]. However, despite important advances in *T. cruzi* genotyping [28] [14] and population genetics [29], [30], the genomic variation between lineages or individual clones of *T. cruzi* remains largely unexplored.

The haploid genome of *T. cruzi* CL Brener (TcVI) is approximately 55 Mbp in size [31]. Analyses of the sequence revealed a repeat-rich, hybrid genome, with long regions of conserved synteny to *Leishmania major* [32] and *Trypanosoma brucei* [33]. A strong signature of the putative TcII/TcIII hybridization that gave rise to TcVI remains. As such, CL Brener predominantly comprises two divergent haplotypes, named Esmeraldo-like (TcII) and non-Esmeraldo-like (TcIII) (abbreviated to Esmeraldo and non-Esmeraldo here). The hybrid nature and repetitive content of this genome complicated its assembly, leaving the first *T. cruzi* genome incomplete by comparison to those *L. major* and *T. brucei*. A later effort to place the contigs and scaffolds into predicted chromosomes increased the length of scaffolds, although resolution still requires considerable improvement [34].

We considered the sequencing of a smaller, less repetitive, non-hybrid *T. cruzi* genome to be a sensible approach to improving resolution. Furthermore, an evolutionarily distinct genome, from a DTU with broader host preferences than TcVI, could provide an interesting basis for comparative genomics. Not only are TcI parasites highly divergent from TcVI in ecology and evolution, but typically they have smaller genomes [28], [35], [36], [37] and have relatively low levels of heterozygosity [30]. They are thus the ideal candidate for analysis. Here we describe shot-gun sequencing and partial genome assembly of Sylvio X10/1, originally isolated in 1983 from a male individual in Pará State, Brazil, suffering from acute Chagas disease [38]. Sylvio X10/1 is a common reference strain of TcI and is frequently used in both *in vivo* and *in vitro* experiments [39] [40] [41] [42]. The genomic contigs and sequence reads were subsequently compared to CL Brener. We found that the core gene content of the two *T. cruzi* lineages is highly similar, but that they harbor large differences in repetitive content and sequence, which may have functional and epidemiological implications.

## Materials and Methods

### Accession numbers

This Whole Genome Shotgun project has been deposited at DDBJ/EMBL/GenBank under the accession ADWP00000000. The version described in this paper is the first version, ADWP01000000. The data will also be available at TriTrypDB [43].

### Sequencing, assembly and annotation

*Trypanosoma cruzi* Sylvio X10/1 cells were cultured at 28°C in RPMI liquid medium supplemented with 0.5% (w/v) tryptone, 20 mM HEPES buffer pH 7.2, 30 mM haemin, 10% (v/v) heat-inactivated foetal calf serum, 2 mM sodium glutamate, 2 mM sodium pyruvate and 25 µg/ml gentamycin. Genomic DNA was extracted using the Gentra Puregene Tissue Kit (Qiagen). Sequencing was performed using 454 technology (FLX/Titanium) and sequence assembly was performed *de novo* using the CELERA assembler (v5.4) [44].

Gene prediction and annotation was performed using GeneMarkS (v2.6p) [45] and best reciprocal BLAST hit to CL Brener. Annotations were manually inspected by alignment to CL Brener using Promer [46] and the Artemis Comparison Tool [47]. Gene models were manually added if found to be missing. In cases where genes were disrupted by sequencing errors, all fragments of the genes were annotated. Truncated genes located on contig ends were annotated when possible.

### Gene specific and evolutionary analysis

Individual genes were identified using reciprocal BLASTp and tBLASTn on both assembled and unassembled reads.

Alignments were created using ClustalW and used to call strain-specific differences; both nucleotide differences and insertion-deletion (indel) events. Calculation of dN/dS was carried out using yn00 (PAML, v4.2) [48]. The McDonald-Kreitman test (MK-test), as implemented in BioPerl (v1.6), was used to evaluate protein adaptation [49], using alignments created by transAlign [50] with *T. brucei* used as the outgroup. Synonymous sites were assumed to be neutral while non-synonymous sites were assumed to be deleterious, neutral or confer an advantage. Positive selection was assumed to take place if the number of inter-species non-synonymous changes was greater than the intra-species changes. A contingency table and Fisher's exact test was used to test for significance. The neutrality index (NI = (Pn/Ps)/(Dn/Ds)) was used to test the direction of adaptation, which is expected to be 1 under neutrality, >1 for positive selection and <1 for purifying selection. Using NI, the proportion of adaptive substitutions can be estimated as α  = 1 - NI.

### Multigene family analysis

Sequence reads with similarity to known gene families in CL Brener were analyzed. Initially, homologous genes were collapsed into families using the clustering tool cdhit [51] at a 90% identity threshold. Subsequently clusters were subject to multiple alignments with ClustalW. Profile hidden markov models (pHMM) were created using hmmbuild (v3, with the parameter –symfrac 0), concatenated to a single file and compressed using hmmpress [52]. Sylvio X10/1 and CL Brener reads were translated into the six reading frames and hmmscan (with the parameters –nobias and –nonull2) was used to conduct searches. To make the results comparable to Sylvio X10/1, Sanger reads from CL Brener were cut into smaller pieces before the HMM search was conducted.

### Results and Discussion

We used 454 technology whole genome shot-gun sequencing [53] to produce a partial assembly as well as a read-based analysis of the TcI reference strain Sylvio X10/1 (TcI) genome. We then conducted a comparison to the genome of the reference strain CL Brener (TcVI). This has allowed the first genome-scale analysis of genetic diversity in *T. cruzi*. The architecture of the two genomes was highly similar, composed of large, co-transcribed, gene-dense “core” coding regions, which displayed highly conserved synteny interspersed with regions of repetitive sequence. The draft assembly has good coverage of these gene dense regions, but is more fragmented in repetitive regions due to the technical difficulties associated with accurate assembly of repeat sequences. However, we have complemented this assembly with a read-based analysis. Thus we were able to characterize comparatively the repeated genes in both genomes. The core gene content of the two genomes was virtually the same but we identified abundant nucleotide and amino acid sequence differences. Furthermore, in the comparison between Sylvio X10/1 and CL Brener we found large differences in the proportion of sequence with homology to multigene families. CL Brener was found to have approximately 5.9 Mbp more of haploid sequence related to the DGF, RHS, mucin, MASP, GP63, and transsialidase gene families. The expansion of these gene families underlies most of the genome size difference between Sylvio X10/1 and CL Brener.

### Sequencing and comparative analyses

Genome sequencing of the TcI isolate Sylvio X10/1 was carried out using 454 technology [53], which generated 582 Mbp sequence data (n_reads_ = 1,688,475, Table 1, Figure S1A), where 79 Mbp (n_reads_ = 301,005) corresponded to maxi/mini circles. Sequence assembly resulted in 7092 contigs (N_50_ = 5659 bp) yielding an average coverage of 11x (Figure S1B). Subsequently, contigs from the assembly were aligned to both CL Brener haplotypes [34] which revealed large blocks of synteny, representing the core gene content of these genomes (i.e. excluding repetitive regions). The amount of heterozygosity in the assembly was examined by counting the number of high quality mismatches between aligned reads, which estimated the heterozygosity to be less than 0.08% in the core genome.

**Table 1:** Data comparison Sylvio X10/1 and CL Brener.

	Sylvio X10/1	CL Brener
**Data amount (10^6^ bp)**	582 a	823 b
**LINE content** c	2.12%	2.27%
**LTR content** c	0.45%	0.50%
**Unique ORFs**	0	6
**Intergenic distance** d	500 bp	500 bp

a 454 sequencing.

b Sanger sequencing.

c Identified using RepeatMasker.

d Average intergenic distance.

**Figure 1:**
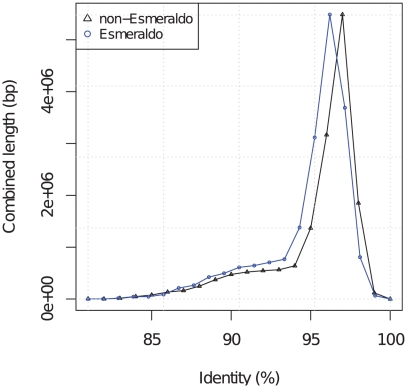
Sequence identity of Sylvio X10/1 contigs compared to non-Esmeraldo and Esmeraldo. Shows the percentage identity (horizontal axis) of the best Sylvio X10/1 versus CL Brener BLAST hit and the combined alignment length on the vertical axis. The black line (triangles) represent Sylvio X10/1 compared to non-Esmeraldo and blue lines (circles) represent Sylvio X10/1 compared to Esmeraldo. Both comparisons have a similar overall distribution of identities but Sylvio X10/1 compared to non-Esmeraldo is shifted to a slightly higher sequence identity. Sylvio X10/1 compared to non-Esmeraldo has a peak at 97% sequence identity and Sylvio X10/1 compared to Esmeraldo has a peak at 96% sequence identity.

**Figure 2:**
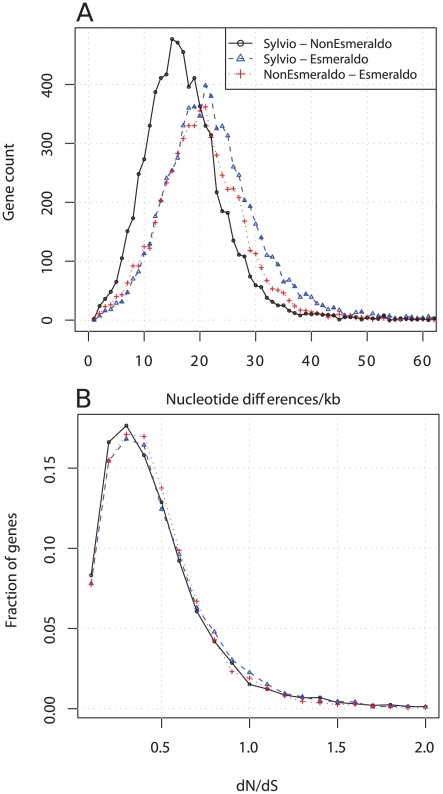
Nucleotide differences and dN/dS estimations. Black lines (circles) represent Sylvio X10/1 compared to non-Esmeraldo, blue lines (triangles) represent Sylvio X10/1 compared to Esmeraldo and red lines (crosses) represent non-Esmeraldo compared to Esmeraldo. A) Shows the distribution of single nucleotide differences (ND) in genes, normalized to show the number of ND per 1000 bp. Sylvio X10/1-Esmeraldo show the largest number of ND, and have 25 ND/kb/gene in average. B) Shows the ratios of non-synonymous and synonymous nucleotide variation (horizontal axis shows dN/dS) between the comparisons as a fraction of the genes examined (vertical axis). All comparisons have average dN/dS around 0.40 and the shape of the curves has a similar appearance. About 95% of the examined genes have a ratio below 1, implying that the genes are under purifying selection and 336 genes show evidence of positive selection (dN/dS >1).

In the coding regions the mean nucleotide identity was higher between Sylvio X10/1 and non-Esmeraldo i.e. TcIII (98.2%) than between Sylvio X10/1 and Esmeraldo i.e. TcII (97.5%) (Table 2, Figure 1 and 2). The mean nucleotide identity between the two CL Brener haplotypes Esmeraldo and non-Esmeraldo was 97.8%. This is independent genome-wide evidence of the generally closer phylogenetic relationship between TcI (Sylvio X10/1) and TcIII (non-Esmeraldo) than with TcII (Esmeraldo). The divergence between these three *T. cruzi* lineages is therefore greater than between *T. brucei* subspecies *T. brucei brucei* and *T. brucei gambiense* (99.2%) [54] but less than between two representatives of different *Leishmania* species complexes, *L. major* and *L. infantum* (94%) [55].

**Table 2:** Sylvio X10/1 compared to the individual haplotypes.

	non-Esmeraldo	Esmeraldo
**Coverage** a	66%	62%
**Nucleotide identity**	98.2%	97.5%
**Nucleotide diversity**	0.0241	0.0310

a Percentage of the chromosomes that are covered by Sylvio X10/1 contigs.

From the alignments, a total of 77,349 putative fixed differences were identified in the coding regions of a total of 5582 genes (8.6 Mbp of sequence). Of these nucleotide differences 52% were synonymous changes, 34% were non-synonymous changes giving rise to chemically similar amino acids and 23% were non-synonymous changes associated with radical amino acid replacement. The average rate of nucleotide differences (ND) between Sylvio X10/1 and non-Esmeraldo was 18 ND/kb/gene and compared to Esmeraldo 25 ND/kb/gene (Figure 2A). In comparison, the average ND rate between non-Esmeraldo and Esmeraldo was 22 NT/kb/gene. This large number of nucleotide differences is consistent with independent evolution of the *T. cruzi* lineages over several million years [27], presumably due to ecological, geographic, and/or reproductive isolation, limiting homogenising forces that might act between lineages. Some of these changes may be adaptive, although one explanation for the high proportion of radical amino acid replacements might be low rates of sexual recombination in *T. cruzi* leading to the accumulation of mildly deleterious mutations over time (Muller's ratchet). Experimental phenotypic comparisons and associated in depth annotation of the potential functional implications of such radical amino acid changes may reveal biological consequences. Multiple CL Brener genes originally thought to have a frame shift not observed in Sylvio X10/1 (n = 169, Table S1) must now also be considered in such comparisons, because our alignments and confirmatory Sanger sequencing revealed they had been mis-assembled and incorrectly annotated as pseudogenes in CL Brener.

Nucleotide substitutions between CL Brener and Silvio X10/1 were not the only coding variations present. A search was also conducted to identify indel events. We identified 1861 coding indels dispersed in 1271 genes. The majority (n = 1350, 72.5%) were caused by length variation in microsatellite tracts. Indels 3 bp in length were the most common, followed by 6 and 9 bp. Multiple genes with a functional annotation (i.e. non-hypothetical genes) were found to contain indels, for example DNA topoisomerase genes, helicase genes, various metabolic genes and chaperones. Several functionally important genes contained relatively large indels, including the DNA repair protein BRCA2, which was found to contain a 44 codon N-terminal deletion in Sylvio X10/1 spanning amino acids 82–125. Although this deletion did not directly affect an evolutionarily conserved domain, it may have functional consequences for BRCA2-mediated homologous recombination capacity in this strain. Deletions were slightly more prevalent in Sylvio X10/1, which could possibly indicate reductive evolution in Sylvio X10/1, or, conversely, that sequence expansion has generally been more common in CL Brener. Similarly, the number of 195 bp satellite repeats was greater in CL Brener [56] [36] and the sum of total intergenic distances was marginally larger in CL Brener (Table 1). The overall content of retroelements, LINEs and LTRs, assessed across both genomes using RepeatMasker and conducted using reads, showed little variation (Table 1).

The clear size differences between the CL Brener and Sylvio X10/1 genomes were confirmed at the macro level. The Sylvio X10/1 haploid genome size was estimated to be 44 Mbp, using extrapolation from the combined length of the contigs from the Sylvio X10/1 assembly (23 Mb) and the unassembled data from repetitive regions (see following sections). Our estimate tallies with previous studies that have estimated the Sylvio X10 genome size at about 35–44 Mbp, using pulse-field gel electrophoresis [37] and flow cytometry [28]. This value for haploid genome size is considerably lower than that for CL Brener (∼55 Mbp) [31]. The smaller genome size appears to be a general feature of TcI strains [28].

### Genome-specific sequences and evolutionary analyses

We found that Sylvio X10/1 and CL Brener have nearly the same core gene complement, including housekeeping genes, structural genes and genes of unknown function. Six annotated open reading frames (ORFs) in CL Brener were not found in Sylvio X10/1 (Table S2). As these ORFs were short (<350 aa) and without a functional annotation, it is unclear whether they are expressed at all. We were not able to identify any Sylvio X10/1-specific genes or significantly long ORFs. However, we note that minimal gene differences are also reported between *T. brucei* subspecies genomes [54], as well as between those of *Leishmania* species [55]. A similar trend has been observed in *Giardia lamblia* [57], [58]. Instead, the great majority of genetic differences between strains of all these parasite genera consist of SNPs and indels as well as, crucially, copy number (see following section).

In the absence of strain specific genes in our dataset, we also screened for those genes that might be under directional selection between Silvio X10/1 and CL Brener. dN/dS ratios (ω) identified 336 genes under positive selection (ω >1), a significant proportion of which (145) were unique to *T. cruzi* by comparison to *T. brucei* and *Leishmania*. The presence of these rapidly evolving *T. cruzi* specific genes could indicate important biological roles in American trypanosomes, for example, genes regulating interactions with hosts or vectors. Those genes that could be assigned function included two genes encoding cell-surface targeted proteins, one 90 kDa surface protein gene and one member of the TolT family. MK tests (see Materials and Methods) for adaptive selection between *T. cruzi* and *T. brucei* identified other genes of known function and putative importance including transporters and various other membrane coupled proteins, as well as, surprisingly, some DNA repair proteins, chaperones and cyclins (Table S2).

### Estimation of multigene family content

**Figure 3:**
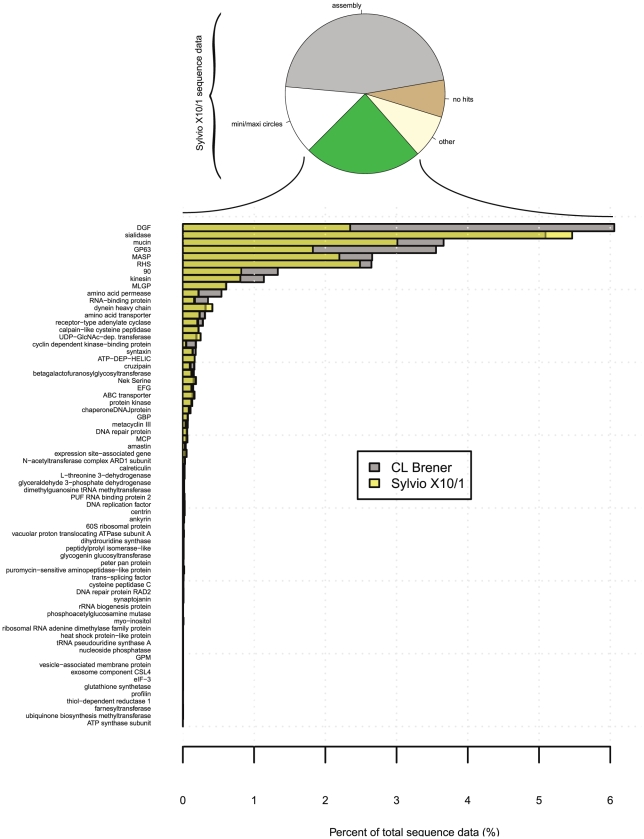
Gene content comparison between Sylvio X10/1 and CL Brener. Shows estimations of gene content between Sylvio X10/1 and CL Brener as percent of the total data. Searches was performed on the read libraries of Sylvio X10/1 and CL Brener. DGF, mucin, MASP, GP63, RHS, 90 and kinesin are more expanded in CL Brener. The sialidase family is indicated to be slightly smaller in Sylvio X10/1.

Many surface proteins involved in interaction with the host in *T. cruzi* are encoded by large repetitive gene families [31]. These regions represent a major area of interest for comparisons between CL Brener and Sylvio X10/1 genomes. Assembly of such repetitive sequences is problematic, therefore we applied a novel approach. The Sylvio X10/1 assembly contained only about 49% of the generated sequence data, leaving 710,109 reads (∼236 Mbp) that did not enter the assembly. To evaluate these data, sequence reads were classified into pre-defined categories using profile hidden markov models. The size of each gene family was estimated using the combined alignment length and normalized to the total amount of sequence data (Figure 3). To provide an estimate of the relative repeat abundance, the same searches were performed on the CL Brener sequence data. To verify the applied method, several single copy genes were included in the analysis. The vast majority of the expected single copy genes resulted in a 1∶1 signal, indicating that the method can be used reliably for copy number quantification.

By this classification approach, a total of 346,696 (49%, 137 Mbp) unused reads from Sylvio X10/1 were sorted into 69 different categories (Figure 3). From these unused reads, 233,574 (33%, 92 Mbp) were assigned to six categories only (sialidase, DGF, RHS, mucin, MASP and GP63). In terms of combined alignment length, these gene families were estimated to represent 7–8 Mbp of the haploid Sylvio X10/1 genome. For Sylvio X10/1 and CL Brener, the sialidase and DGF categories were the largest for each genome respectively, comprising 5.4% and 6.1% of the sequence data. According to this analysis, a smaller proportion of the sequence reads match the DGF family in Sylvio X10/1, suggesting that this family is expanded in CL Brener or contracted in Sylvio X10/1. The analysis also indicated copy number differences for the MASP, mucin, GP63 and RHS gene families between the two genomes. It should be noted that this method does not discriminate between pseudogenes and functional genes and therefore, some of the predicted genes could represent non-functional or non-expressed gene variants.

In addition to inter-genomic comparisons between the major gene families, a more comprehensive analysis was performed on a larger set of *T. cruzi* genes, which included 5874 different homologous gene clusters, including singletons. The most significant differences were found among some hypothetical genes, and in most cases there was an expansion in CL Brener.

These comparative analyses of both the non-coding and coding repetitive elements indicates significant differential expansion in sequence corresponding to surface antigen repertoires and other multicopy gene families. The CL Brener genome was estimated to have about 5.9 Mbp (11.8 Mbp diploid) of extra sequence related to multigene families than Sylvio X10/1. Therefore, we conclude that expanded gene families in CL Brener underlie most of the genome size difference between TcI and TcVI, and this may theoretically enhance functional plasticity. CL Brener (TcVI) is the product of hybridization between TcII and TcIII [59]. We cannot determine whether the gene family expansions occurred pre- or post-hybridisation (or both). However, TcII, TcIII and TcVI strains all have similarly increased DNA contents relative to TcI [28]. This suggests the bulk of expansion occurred within ancestral TcII and TcIII.

### Conclusions

This first intra-species comparative genomic analysis of *T. cruzi* provides several significant insights. First, it is clear that core genome synteny and gene identity are highly conserved between TcI and TcVI, with very few unique and no major gene differences. Similarly, the overall quantity of non-coding DNA is largely unchanged between the two genomes. The most significant variation between the two genomes is in the size of several multigene families, which encode many important surface proteins. These families are significantly larger in TcVI and account for approximately 54% of the c.11 Mbp size difference between TcVI and TcI. Our findings compare well with recent comparative genomic studies of other parasitic trypanosomes at the sub-species (*T. brucei*, [54]) and species complex (*Leishmania*, [55]) level. In both cases few gene differences are apparent in the core genomes, congruent with the remarkable synteny observed at the inter-species level [31]. This apparent lack of genomic rearrangement, gene deletion and insertion between trypanosome genomes could derive from the constraints of polycistronic transcription, disruptions of these long co-transcribed gene clusters being likely to be deleterious.

Genetic recombination is a common mechanism by which structural change may be introduced between genomes, as well are providing sources of new genetic information. The excessive accumulation of non-synonymous changes that we observe between TcI and TcVI suggest that this recombination may be infrequent in *T. cruzi* at the inter-DTU level at least. However, the overall natural frequency of intra-species and intra-genotype genetic recombination in all three major human parasitic trypanosome genera is a still a matter of some uncertainty and considerable debate [60], [61], [62], [63], [64], [65]. Functional dissection of the larger surface gene families in TcVI presents an interesting problem. Both TcI and TcVI efficiently infect humans and TcVI is found among far fewer hosts than TcI [3]. However, TcVI may have emerged quite recently in conjunction establishment in the human host (Lewis et al, submitted). It remains to be defined how much of the differential surface gene diversity is actually expressed.

This study represents a significant advance in unraveling the diversity of *T. cruzi* and encourages further comparative genomics of the *T. cruzi* lineages and related species of the subgenus *Schizotrypanum*. We are currently engaged in sequencing other representatives of TcI, and the apparently bat specific trypanosome *T. cruzi marinkellei*.

## Supporting Information

Figure S1 **Read lengths and assembly coverage.** A) Shows the number of reads (vertical axis) with a certain read length in base pairs (horizontal axis). Shaded lines (light grey) represent 454 FLX reads and black lines represent 454 Titanium reads. One major peak at around 250 bp is distinct for FLX and another major peak at 500 bp is distinct for the 454 Titanium. B) Shows the coverage of the assembly. Number of positions is plotted on the vertical axis with a certain level of redundancy (coverage) on the horizontal axis. The curve has a peak at 11 times coverage, which is the mean coverage.
(0.10 MB PDF)

Table S1 **Incomplete genes and incorrect pseudogenes in CL Brener.** An Excel file containing three sheets: A) Genes in Esmeraldo (CL Brener) with sequence gaps and the corresponding ortholog in Sylvio X10/1. B) Genes in non-Esmeraldo (CL Brener) with sequence gaps and the corresponding ortholog in Sylvio X10/1. C) Pseudogenes in CL Brener that is likely to contain a sequencing or assembly error in this genome.
(0.10 MB XLS)

Table S2 **Evolutionary analysis and specific genes.** An Excel file containing three sheets: A) Contains dN/dS estimates for Sylvio X10/1 versus Esmeraldo and Sylvio X10/1 versus non-Esmeraldo, along with product descriptions and information about signal peptides and transmembrane domains. B) Contain detailed results from the McDonald-Kreitman test, for those genes that were subject to this analysis. C) A list of CL Brener open reading frames that were not identified in Sylvio X10/1.
(3.37 MB XLS)
