# Supplementary figures and images for: Shotgun Sequencing Analysis of Trypanosoma cruzi I Sylvio X10/1 and Comparison with T. cruzi VI CL Brener
Source: PLoS Negl Trop Dis. 2011 Mar 8;5(3):e984. doi: 10.1371/journal.pntd.0000984 (PMC3050914; doi:10.1371/journal.pntd.0000984)

**A**

Length distribution of sequence reads

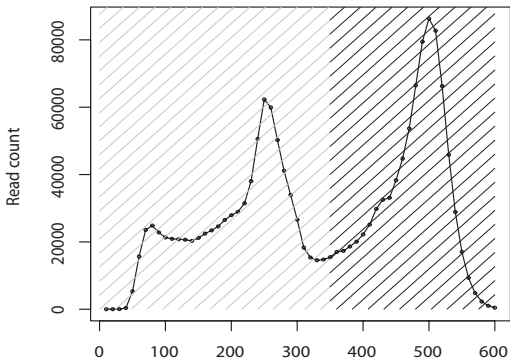**B**Length (bp)  
Assembly coverage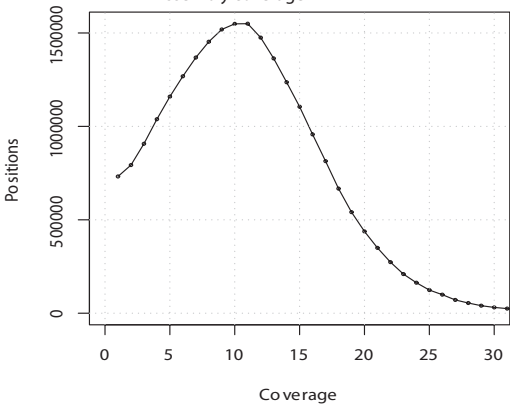

Supplement: Figure S1 — Read lengths and assembly coverage. A) Shows the number of reads (vertical axis) with a certain read length in base pairs (horizontal axis). Shaded lines (light grey) represent 454 FLX reads and black lines represent 454 Titanium reads. One major peak at around 250 bp is distinct for FLX and another major peak at 500 bp is distinct for the 454 Titanium. B) Shows the coverage of the assembly. Number of positions is plotted on the vertical axis with a certain level of redundancy (coverage) on the horizontal axis. The curve has a peak at 11 times coverage, which is the mean coverage. (0.10 MB PDF) [file pntd.0000984.s001.pdf]
